# Authenticity and Bioactive Markers Search in the Phenolic‐Rich Extracts of Asteraceae Medicinal Plants Through Integrative Computational Chemometrics

**DOI:** 10.1002/fsn3.4720

**Published:** 2025-01-17

**Authors:** Pascual Garcia‐Perez, Paula Garcia‐Oliveira, Tiane C. Finimundy, Jose Pinela, Ricardo C. Calhelha, Marija Nenadić, Marina Soković, Jesus Simal‐Gandara, Lillian Barros, Miguel A. Prieto

**Affiliations:** ^1^ Department of Food Technology, Nutrition and Food Science Veterinary Faculty, University of Murcia Murcia Spain; ^2^ Nutrition and Bromatology Group, Department of Analytical Chemistry and Food Science, Instituto de Agroecoloxía e Alimentación (IAA) – CITEXVI Universidade de Vigo Vigo Spain; ^3^ CIMO, LA SusTEC Instituto Politécnico de Bragança Bragança Portugal; ^4^ Institute for Biological Research “Siniša Stanković” – National Institute of Republic of Serbia University of Belgrade Belgrade Serbia

**Keywords:** antioxidants, functional analysis, multivariate statistics, nutraceuticals, regularized canonical component analysis

## Abstract

The Asteraceae family has been of significant concern for ethnobotanical studies, thanks to its health‐promoting properties linked to a plethora of bioactive compounds, among which phenolic compounds play a critical role. In this work, a workflow based on computational chemometrics was employed to assess the authenticity and biomarker search of five key Asteraceae species commonly employed in traditional medicine. The UHPLC‐DAD‐ESI/MS–MS phenolic profile of Asteraceae extracts was combined with the evaluation of several in vitro biological properties. Caffeoylquinic acids (CQAs), chicoric acids, and flavonoid glycosides were reported as authenticity markers of *Achillea millefolium* , *Taraxacum officinale* , and *Arnica montana* , respectively. The integration of phenolic profile and in vitro bioactivities provide insights for the identification of *trans* 3,5‐*O*‐dicaffeoylquinic acid (3,5‐*O*‐diCQA) and isorhamnetin glycosides as the major antioxidant agents in Asteraceae extracts, whereas several CQAs and caffeoyl‐deoxy‐octulopyranosonic acids have been reported as responsible for their cytotoxic and anti‐inflammatory activities. These results shed light on the authentication and quality evaluation of Asteraceae extracts, along with the characterization of their functional properties, leading to their application in the design of novel plant‐based functional foods.

## Graphical abstract

The phenolic profiling and in vitro bioactivities of five Asteraceae species were evaluated. *Achillea millefolium* and *Arnica montana* stand out as rich sources of phenolic compounds, which correlate with their significant antioxidant results. *Chamaemelum nobile* contained a high amount of flavonoids, which may be involved in its anti‐inflammatory effects. Finally, *Calendula officinalis* revealed strong antimicrobial effects. The integration of datasets identified diverse compounds as antioxidant, anti‐inflammatory, and cytotoxic agents.

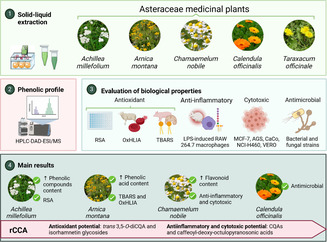

## 1 Introduction

Plants have been widely used by humans for multiple purposes, including nutrition, ornamentation, cosmetics, and as natural remedies for the treatment of different diseases in traditional medicine (Srivastava 2018). In this case, medicinal plants have gained significant attention in the field of pharmacological research, as they are considered natural sources of phytochemicals with valuable bioactivities, as a result of their secondary metabolism (Ceylan et al. 2021). In parallel, such plant bioactive compounds have also attracted the attention of other economically important sectors, as these compounds can be incorporated as functional ingredients in the development of either nutraceutical food products or cosmetic preparations with enhanced properties (Patra et al. 2018; Yilmaz 2020). Among the wide range of plant species widely known for their biological activities, the Asteraceae family stands out as a relevant plant family, which contains around 24,000 reported species classified into 1600 different genera, making it one of the largest families within the plant kingdom. This family is ubiquitously distributed across all regions except Antarctica (Michel, Abd Rani, and Husain 2020). Some Asteraceae species have been employed as medicinal plants in the traditional medicine of different cultures worldwide, being part of different formulations, including decoctions, infusions, ointments, and lotions for the treatment of both acute and chronic diseases (Garcia‐Oliveira et al. 2021; Yener et al. 2020).

Based on this, a large number of Asteraceae species have been characterized by their promotion of different biological properties, acting as powerful sources of antioxidant, anti‐inflammatory, analgesic, anticancer, and antimicrobial agents (Garcia‐Oliveira et al. 2021; Jayasundera et al. 2021). Such bioactivities have been attributed to the biosynthesis of bioactive compounds. In the case of the Asteraceae family, a plethora of bioactive compounds of different biosynthetic origins has been reported, including not only the ubiquitously found phenolic compounds but also polysaccharides, terpenoids, fatty acids, phytosterols, and alkaloids (Bessada, Barreira, and Oliveira 2015). Among them, phenolic compounds have been pointed out as the major phytoconstituents in Asteraceae species responsible for their associated bioactivities, thanks to their multifaceted effects as bioactive compounds (Petropoulos et al. 2019). Phenolic compounds exhibit different health‐enhancing properties, as demonstrated at different levels, from in vitro and in vivo studies to interventional clinical trials in humans (Villegas‐Aguilar et al. 2022), thus gaining significant attention in recent years as natural additives in food, pharmacological, and cosmetic preparations, with the aim of adding value to such products (Albuquerque et al. 2021). However, there is a gap in knowledge regarding the authenticity of Asteraceae species, as well as in the mechanism of action of their bioactive compounds. The determination of interactive phenomena between their phytoconstituents is usually underestimated, resulting in information derived from poorly characterized raw extracts.

In this work, five commercial products intended for consumption as infusions based on species belonging to the Asteraceae family, largely used in the traditional medicine of Galicia (North‐West Spain), were evaluated: *Achillea millefolium* , *Calendula officinalis* , *Taraxacum officinale* , *Arnica montana* , and *Chamaemelum nobile* . Currently, these species are marketed, mainly as infusions, different herbal food formulations, and other products. The industrial use of these species could be expanded by extracting their bioactive compounds and obtaining highly bioactive extracts. Considering possible applications, especially in the food sector, water–alcohol mixtures are commonly used for phenolic compound extraction. Therefore, this study had two main objectives: (1) the evaluation of the authenticity biomarkers of ethanolic extracts of selected species to assess their quality and avoid fraud and adulteration; and (2) the determination of metabolite markers responsible for their functional traits, contributing to the valorization of these extracts. For this purpose, Asteraceae extracts were subjected to phenolic profiling through an HPLC‐DAD‐ESI/MS–MS analytical approach. In addition, several in vitro properties were evaluated, including antioxidant, anti‐inflammatory, anticancer, cytotoxic, and antimicrobial activities. Moreover, Asteraceae extracts were explored using multivariate statistics and chemometrics‐based data integration to search for relevant bioactive markers responsible for the reported biological activities and eventual synergistic and/or antagonistic relationships between their phenolic phytoconstituents. This novel computational approach will contribute to featuring phytochemical candidates of Asteraceae medicinal species, paving the way for their industrial exploitation by different sectors.

## 2 Materials and Methods

### 2.1 Plant Material

Five Asteraceae species were selected in this work, namely: *T. officinale* F. H. Wigg (TO), *C. officinalis* L. (CO), *A. millefolium* L. (AC), *A. montana* L. (AR), and *C. nobile* (L.) All. (CN). Dried flowers of AR, CO, and CN, and dried leaves, stems, and flowers of AC and TO were purchased from Soria Natural (Soria, Spain; www.sorianatural.es) and Pinisan (Madrid, Spain; www.pinisan.com).

### 2.2 Preparation of Plant Extracts and Phenolic Profile

Samples were air‐dried, crushed by a blender, and further sieved to obtain a fine homogeneous powder. One gram of each dry sample was weighed, dissolved in 30 mL of the extraction solvent, which consisted of 80% aqueous ethanol (*v/v*), and stirred magnetically for 1 h in darkness to obtain plant extracts. Samples were then filtered and subjected to rotary evaporation at 40°C. The remaining fraction was later lyophilized until dry extracts were obtained.

Asteraceae extracts were resuspended with 80% EtOH (v/v) at 10 mg/mL, syringe‐filtered (0.2 μm) into analytical vials, and subjected to phenolic profiling via ultrahigh‐performance liquid chromatography‐diode array detection coupled to tandem mass spectrometry through electrospray ionization (UHPLC‐DAD‐ESI/MS^2^) approach. For this purpose, the chromatographic system used was a Dionex Ultimate 3000 UHPLC system (Thermo Scientific, USA) equipped with a Spherisorb S3 ODS‐2C_18_ column (3 μm, 4.6 mm × 150 mm; Waters, USA), and a DAD set at 280, 330, and 370 nm. MS was performed using an ESI‐Linear Ion Trap LTQ XL MS (Thermo Finnigan, USA) in negative mode, set in full‐scan acquisition mode, with a 100–1500 *m/z* range. The rest of the operational conditions were previously optimized and described elsewhere, using the Xcalibur software (Thermo Finnigan) for data acquisition (Bessada et al. 2016). When possible, phenolic compounds were identified by comparing their retention times, UV–vis, and mass spectra with those of commercial standards: chlorogenic acid, caffeic acid, quercetin‐3‐*O*‐glucoside, naringenin, apigenin 6‐*C*‐glucoside and apigenin 7‐*O*‐glucoside, obtained from Extrasynthese (Genay, France). If commercial standards were not available, compounds were tentatively identified by comparing the obtained information with previous literature. For quantification, a calibration curve was created for each available phenolic standard available, based on the UV signal. Data for the calibration curves are provided in Table 1. For identified phenolic compounds without a commercial standard, quantification was performed using the calibration curve of the most similar available standard. The results were expressed as mg per g of dry extracts (mg/g DW), and determinations were carried out in triplicate.

### 2.3 Evaluation of In Vitro Biological Activities

Asteraceae extracts were subjected to in vitro biological activity evaluation, following the corresponding protocols previously optimized (Pereira et al. 2023). Antioxidant activity was determined by free‐radical scavenging activity (RSA), lipid peroxidation inhibition (TBARS), and oxidative hemolysis inhibition (OxHLIA), and the results were expressed as inhibitory concentration (IC_50_) in μg/mL of Asteraceae extracts, using Trolox as a positive control. Cytotoxic activity of Asteraceae extracts was determined using four human tumor cell lines, namely: AGS, Caco‐2, MCF‐7, and NCI‐H460 (gastric adenocarcinoma, colorectal adenocarcinoma, breast adenocarcinoma, and non‐small cell lung cancer cell lines, respectively). Moreover, a non‐tumor cell line VERO (monkey kidney fibroblasts) was selected to evaluate the cytotoxicity of plant extracts toward healthy cells, and the results were presented as growth inhibitory concentration 50 (GI_50_) in μg/mL of Asteraceae extracts using ellipticine as a positive control. The anti‐inflammatory activity was determined by means of nitric oxide (NO) production inhibition by lipopolysaccharide (LPS)‐induced murine macrophages RAW 264.7 cell line, and the results were expressed as (IC_50_) in μg/mL of Asteraceae extracts, using dexamethasone as a positive control. The antimicrobial activity of Asteraceae extract was performed by the microdilution method (Sokovicx́ et al. 2010), against different Gram‐positive bacteria: *Staphylococcus aureus* , *Bacillus cereus* , and *Listeria monocytogenes* (ATCC 11632, clinical isolate, and NCTC 7973, respectively); and also Gram‐negative bacteria: *Escherichia coli* , *Salmonella typhimurium* , and *Enterobacter cloacae* (ATCC 25922, ATCC 13311, and ATCC 35030, respectively). Antifungal activity was tested against six different pathogenic species, that is, *Aspergillus fumigatus* , *Aspergillus niger*, *Aspergillus versicolor*, *Penicillium funiculosum*, *Trichoderma viride*, and *Penicillium verrucosum* var. *cyclopium* (human isolate, ATCC 6275, ATCC 11730, ATCC 36839, IAM 5061, and food isolate, respectively). The results were expressed as mg/mL of Asteraceae extracts by means of minimal inhibitory concentration (MIC) in both cases, minimal bactericidal concentration (MBC) for antibacterial activity, and minimal antifungal concentration (MFC) for antifungal activity. Sodium benzoate (E‐211) and potassium metabisulfite (E‐224), two synthetic and widely used preservatives, were used as positive controls.

### 2.4 Statistical and Multivariate Analysis

The results for all determinations were performed in three independent experimental replicates (*n* = 3) and expressed as the mean value ± standard deviation (SD). Statistical analysis was performed using the software SPSS 25 (IBM Corp., 2017, Armonk, NY, USA). One‐way analysis of variance (ANOVA) statistically assessed the differences between Asteraceae extracts, followed by Tukey's post hoc test, setting a significance threshold of *α* = 0.05. Student's *t*‐test was applied to assess statistically significant differences involving pairwise comparisons (*α* = 0.05).

The multivariate statistical analysis of Asteraceae phenolic profiling was performed using the online platform MetaboAnalyst 5.0 (available at metabolyst.ca). For this purpose, the concentrations of phenolic compounds were incorporated into a unique dataset, normalized by the median, and auto‐scaled. Data were then analyzed by means of unsupervised clustering (Euclidean distance measurement, Ward's clustering algorithm), and principal component analysis (PCA), as well as by supervised partial least squares (PLS) analysis, providing the most discriminant features through the determination of VIP (variable importance in projection) markers, setting a VIP score = 1 cut‐off.

### 2.5 Data Integration Through Regularized Canonical Component Analysis (rCCA)

The datasets obtained from the collected phenolic profile and in vitro biological activities were integrated by rCCA, using the MixOmics package and Pearson correlation in R (v.4.3.2.) (Rohart et al. 2017). The rCCA model was obtained following a previous optimization by cross‐validation tuning to maximize the correlations between both datasets. The results were displayed through a sample plot, indicating the projection of the different samples led by the canonical variates, along with a correlation structure network (correlation cut‐off = 0.5) that reflects the relationship between significantly correlated features.

## 3 Results and Discussion

### 3.1 Phenolic Profile of Asteraceae Extracts

**FIGURE 1:**
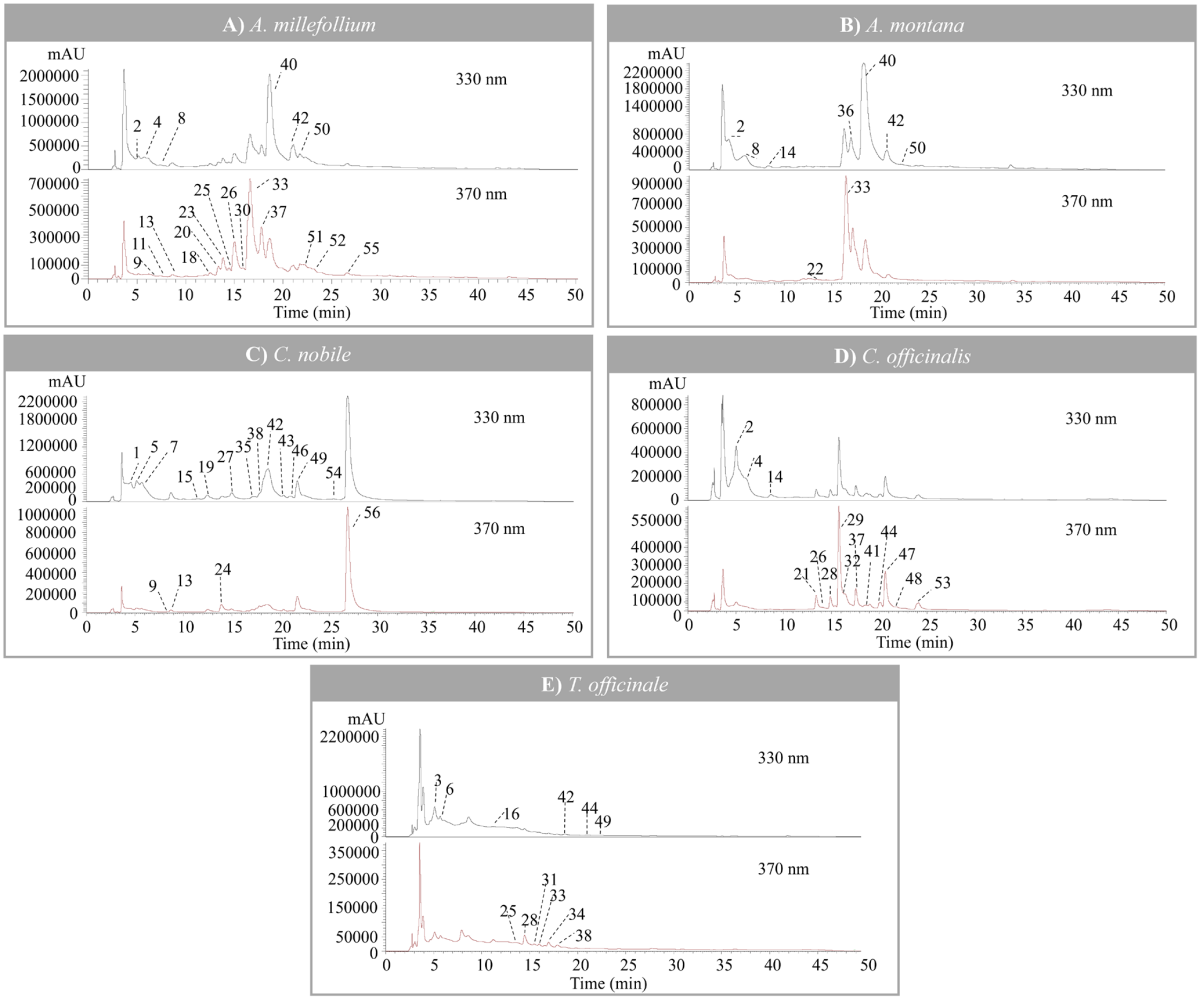
Representative phenolic profile of Asteraceae extracts through UHPLC‐DAD‐ESI/MS^2^, recorded at 330 and 370 nm. For peak numbers, refer to Table 1.

The characterization of phenolic compounds in the five Asteraceae species was performed by HPLC‐DAD‐ESI/MS^2^. The identified compounds are reported in Table 1, combined with a representative chromatogram of each species shown in Figure 1.

**TABLE 1:** Retention time (Rt), wavelengths of maximum absorption in the visible region (*λ*_max_), mass spectral data, identification, and quantification of phenolic compounds in *Taraxacum officinalis*, *Calendula officinalis* , *Achillea millefolium* , *Arnica montana* , and *Chamaemelum nobile* (mean&amp;#x02009;±&amp;#x02009;SD; mg/g extract).

ID	Rt	*λ*_max_	[M‐H]^−^	MS^2^	Tentative identification	Plant species				
	(min)	(nm)	(m/z)	(m/z)		*Taraxacum officinalis*	*Calendula officinalis*	*Achillea millefolium*	*Arnica montana*	*Chamaemelum nobile*
1	4.50	323	381	337 (5), 293 (12), 251 (6), 245 (31), 179 (28), 161 (100), 135 (8)	3‐CDOA	n.d.	n.d.	n.d.	n.d.	1.03 ± 0.01
2	4.55	324	353	191 (100), 179 (79), 135 (3)	*cis*‐3‐*O*‐Caffeoylquinic acid	n.d.	9.8 ± 0.2b	19.7 ± 0.5a	3.7 ± 0.1c	n.d.
3	5.11	324	353	191 (100), 179 (23), 135 (3)	*trans*‐3‐*O*‐Caffeoylquinic acid	6.7 ± 0.42	n.d.	n.d.	n.d.	n.d.
4	5.15	323	353	191 (70), 179 (45), 173 (100), 135 (5)	4‐*O*‐Caffeoylquinic acid	n.d.	5.2 ± 0.2*	3.21 ± 0.02	n.d.	n.d.
5	5.17	325	381	363 (15), 337 (12), 293 (42), 251 (52), 203 (13), 179 (51), 161 (100), 135 (7)	4‐CDOA	n.d.	n.d.	n.d.	n.d.	0.77 ± 0.05
6	5.72	320	341	179 (100)	Caffeic acid hexoside	1.9 ± 0.1	n.d.	n.d.	n.d.	n.d.
7	5.76	324	381	337 (7), 293 (25), 251 (34), 245 (5), 179 (20), 161 (100), 135 (6)	9‐CDOA	n.d.	n.d.	n.d.	n.d.	1.8 ± 0.1
8	5.93	325	353	191 (100), 179 (19), 173 (6), 135 (4)	5‐*O*‐Caffeoylquinic acid	n.d.	n.d.	3.61 ± 0.05	20.3 ± 0.3*	n.d.
9	7.45	322	431	269 (100)	Apigenin‐7‐*O*‐glucoside	n.d.	n.d.	6.3 ± 0.4*	n.d.	5.2 ± 0.2
10	7.92	323	473	311 (100)	*cis*‐Chicoric acid	0.53 ± 0.02	n.d.	n.d.	n.d.	n.d.
11	7.97	319	573	503 (25), 473 (100), 383 (52), 353 (44)	Apigenin‐6,8‐di‐*C*‐hexoside isomer 1	n.d.	n.d.	2.6 ± 0.1	n.d.	n.d.
12	8.60	322	473	311 (100)	*trans*‐Chicoric acid	2.9 ± 0.2	n.d.	n.d.	n.d.	n.d.
13	8.61	323	593	505 (14), 473 (24), 383 (18), 353 (29), 325 (11)	Apigenin‐6,8‐di‐*C*‐hexoside isomer 2	n.d.	n.d.	2.0 ± 0.1	n.d.	19 ± 1*
14	8.66	322	179	135 (100)	Caffeic acid	n.d.	0.83 ± 0.03b	n.d.	2.3 ± 0.1a	n.d.
15	11.09	320	705	543 (100)	3,9‐di‐CDOA glucoside	n.d.	n.d.	n.d.	n.d.	0.40 ± 0.02
16	11.18	323	473	311 (100), 293 (25)	Caffeoyl hexosyl‐pentoside	0.30 ± 0.02	n.d.	n.d.	n.d.	n.d.
17	11.19	317	367	191 (100), 193 (8)	5‐*O*‐Feruloylquinic acid	n.d.	1.22 ± 0.03*	n.d.	0.76 ± 0.03	n.d.
18	12.41	325	563	473 (30), 413 (100), 383 (8), 341 (28), 313 (15), 293 (48)	Apigenin 2″‐*O*‐pentosyl‐6‐*C*‐hexoside	n.d.	n.d.	4.1 ± 0.1	n.d.	n.d.
19	12.52	321	839	677 (32), 515 (100), 353 (8), 341 (15), 191 (5), 179 (9)	1,3,4,5‐Tetra‐*O*‐caffeoylquinic	n.d.	n.d.	n.d.	n.d.	0.91 ± 0.03
20	13.25	337	447	357 (100), 327 (88), 297 (18), 285 (19)	Luteolin 6‐*C*‐glucoside	n.d.	n.d.	2.4 ± 0.1	n.d.	n.d.
21	13.42	350	755	301 (100)	Quercetin‐3‐*O*‐deoxyhexosyl‐rutinoside	n.d.	0.93 ± 0.03	n.d.	n.d.	n.d.
22	13.49	320	463	287 (100)	Eriodictyol‐*O*‐glucuronide	n.d.	n.d.	n.d.	2.2 ± 0.1	n.d.
23	13.79	339	447	285 (100)	Luteolin‐*O*‐hexoside	n.d.	n.d.	2.60 ± 0.02	n.d.	n.d.
24	13.84	356	479	317 (100)	Myricetin‐3‐*O*‐glucoside	n.d.	n.d.	n.d.	n.d.	1.60 ± 0.03
25	14.17	334	609	285 (100)	Kaempferol‐di‐*O‐*hexoside	0.87 ± 0.03	n.d.	2.3 ± 0.1*	n.d.	n.d.
26	14.90	350	609	301 (100)	Quercetin‐3‐*O*‐rutinoside	0.578 ± 0.001c	0.86 ± 0.03b	1.4 ± 0.1a	n.d.	n.d.
27	15.01	320	677	515 (28), 497 (16), 353 (65), 335 (23), 191 (12), 179 (8), 135 (3)	1,3,5‐*O* or 1,4,5‐*O*‐Tricaffeoylquinic	n.d.	n.d.	n.d.	n.d.	2.3 ± 0.1
28	15.42	354	623	461 (100), 315 (51)	Isorhamnetin‐*O*‐deoxyhexosyl‐hexoside	0.63 ± 0.01	n.d.	n.d.	n.d.	n.d.
29	15.80	354	769	315 (100)	Isorhamnetin‐*O*‐deoxyhexosyl‐rutinoside	n.d.	3.3 ± 0.1	n.d.	n.d.	n.d.
30	15.86	332	609	301 (100)	Quercetin‐*O*‐deoxyhexosyl‐hexoside	n.d.	n.d.	6.8 ± 0.1	n.d.	n.d.
31	16.00	327	577	487 (46), 473 (10), 457 (100), 383 (5), 353 (26), 311 (25)	Apigenin‐*C*‐hexoside‐*C*‐rhamnoside	0.62 ± 0.04	n.d.	n.d.	n.d.	n.d.
32	16.44	352	769	315 (100)	Isorhamnetin‐3‐*O*‐deoxyhexosyl‐rutinoside	n.d.	1.2 ± 0.1	n.d.	n.d.	n.d.
33	16.55	351	477	301 (100)	Quercetin‐3‐*O*‐glucuronide	n.d.	n.d.	5.5 ± 0.1	5.4 ± 0.3	n.d.
34	16.98	350	593	285 (100)	Luteolin‐*O*‐deoxyhexosyl‐hexoside	1.4 ± 0.1	n.d.	n.d.	n.d.	n.d.
35	17.03	328	543	381 (65), 363 (18), 319 (7), 221 (18), 179 (59), 161 (50), 135 (25)	4,9‐di‐CDOA	n.d.	n.d.	n.d.	n.d.	0.77 ± 0.03
36	17.15	339	515	353 (100), 335 (13), 191 (13), 179 (25), 173 (42)	3,4‐*O*‐Dicaffeoylquinic acid	n.d.	n.d.	n.d.	15 ± 1	n.d.
37	17.71	345	623	315 (100)	Isorhamnetin‐3‐*O*‐deoxyhexosyl‐hexoside isomer 1	n.d.	1.4 ± 0.1	4.1 ± 0.1*	n.d.	n.d.
38	17.80	324	543	381 (62), 251 (6), 221 (24), 203 (16), 179 (83), 161 (82), 135 (42)	3,9‐di‐CDOA	n.d.	n.d.	n.d.	n.d.	0.98 ± 0.05
39	17.88	348	447	285 (100)	Luteolin‐7‐*O*‐glucoside	1.2 ± 0.1	n.d.	n.d.	n.d.	n.d.
40	18.53	326	515	353 (100), 191 (78), 179 (66), 161 (5), 135 (21)	*cis*‐3,5‐*O*‐Dicaffeoylquinic acid	n.d.	n.d.	43 ± 1	45 ± 1	n.d.
41	18.60	332	505	301 (100)	Quercetin‐3‐*O*‐acetylhexoside	n.d.	0.75 ± 0.01	n.d.	n.d.	n.d.
42	18.63	328	515	353 (100), 191 (32), 179 (81), 135 (18)	*trans*‐3,5‐*O*‐Dicaffeoylquinic acid	0.51 ± 0.02c	n.d.	10.9 ± 0.4a	7.1 ± 0.02b	5.8 ± 0.3b
43	18.74	326	543	381 (100), 363 (11), 319 (6), 221 (8), 179 (42), 161 (24), 135 (10)	3,4 di‐CDOA	n.d.	n.d.	n.d.	n.d.	1.2 ± 0.01
44	19.05	338	609	315 (100)	Isorhamnetin‐*O*‐pentosyl‐hexoside	n.d.	0.73 ± 0.001	n.d.	n.d.	n.d.
45	19.84	325	469	307 (8), 179 (100),161 (37), 135 (78)	Caffeoyl‐hexoside‐methylglutarate	0.004 ± 0.0002	n.d.	n.d.	n.d.	n.d.
46	20.28	327	543	363 (27), 337 (15), 319 (6), 293 (50), 251 (99), 221 (4), 179 (100), 161 (99), 135 (14)	di‐CDOA	n.d.	n.d.	n.d.	n.d.	5.3 ± 0.2
47	20.13	346	709	665 (15), 489 (60), 285 (100)	Luteolin‐*O*‐malonylglucuronyl‐hexoside	n.d.	1.20 ± 0.04	n.d.	n.d.	n.d.
48	21.13	354	623	315 (100)	Isorhamnetin‐3‐*O*‐deoxyhexosyl‐hexoside isomer 2	n.d.	0.79 ± 0.01	n.d.	n.d.	n.d.
49	21.34	321	515	353 (90), 191 (88), 179 (22), 173 (72), 135 (58)	*cis*‐4,5‐*O*‐Dicaffeoylquinic acid	0.38 ± 0.02	n.d.	n.d.	n.d.	2.3 ± 0.1*
50	21.66	330	515	353 (100), 191 (2), 179 (17), 173 (40)	*trans* 4,5‐*O*‐Dicaffeoylquinic acid	n.d.	n.d.	5.6 ± 0.2*	1.4 ± 0.1	n.d.
51	22.19	331	533	489 (100), 285 (30)	Luteolin‐*O*‐malonyl‐hexoside	n.d.	n.d.	4.4 ± 0.1	n.d.	n.d.
52	22.96	331	563	473 (12), 443 (25), 383 (18), 353 (31)	Apigenin‐*C*‐hexoside‐*C*‐pentoside	n.d.	n.d.	3.2 ± 0.2	n.d.	n.d.
53	24.05	352	519	315 (100)	Isorhamnetin‐*O*‐acetyl‐hexoside	n.d.	0.85 ± 0.05	n.d.	n.d.	n.d.
54	24.86	326	499	353 (100), 179 (20), 163 (5), 119 (3)	4‐Caffeoyl‐5‐*p*‐coumaroylquinic acid	n.d.	n.d.	n.d.	n.d.	1.2 ± 0.1
55	26.57	329	473	269 (100)	Apigenin‐*O*‐acetyl‐hexoside	n.d.	n.d.	2.01 ± 0.01	n.d.	n.d.
56	26.89	345	579	285 (100)	Luteolin‐*O*‐pentosyl‐hexoside	n.d.	n.d.	n.d.	n.d.	50.0 ± 0.5
					TPA	13 ± 1	17.2 ± 0.3	85.99 ± 0.05	111.0 ± 0.4	24.0 ± 0.1
					TF	5.3 ± 0.2	12.0 ± 0.1	50 ± 1	7.6 ± 0.2	76 ± 1
					TPC	19 ± 1	29.2 ± 0.3	136 ± 1	119 ± 1	100 ± 1

*Note:* Calibration curves: chlorogenic acid (*y* = 168,823*x* − 161,172; *R* ^2^ = 0.9999; LOD = 0.20 μg/mL; LOQ = 0.68 μg/mL), caffeic acid (*y* = 388,345*x* + 406,369; *R* ^2^ = 0.994; LOD = 0.78 μg/mL; LOQ = 1.97 μg/mL), quercetin‐3‐*O*‐glucoside (*y* = 34,843*x* − 160,173, R^2^ = 0.9998; LOD 0.21 μg/mL; LOQ 0.71 μg/mL), naringenin (*y* = 18,433*x* + 78,903; *R* ^2^ = 0.9998; LOD = 0.17 μg/mL; LOQ = 0.81 μg/mL), apigenin 6‐*C*‐glucoside (*y* = 107,025*x* − 61,531; *R* ^2^ = 0.9989; LOD = 0.19 μg/mL; LOQ = 0.63 μg/mL), apigenin 7‐*O*‐glucoside (*y* = 10,683*x* − 45,794; *R* ^2^ = 0.999; LOD = 0.10 μg/mL; LOQ = 0.53 μg/mL). For all phenolic compounds detected in less than three samples, differences among means were compared by Student's *t*‐test. For each species, means within a line with different letters differ significantly (*p* < 0.05).

Abbreviations: CDOA, caffeoyl‐2,7‐anhydro‐3‐deoxy‐2‐octulopyranosonic acid; n.d., not detected; TF, total flavonoids; TPA, total phenolic acids; TPC, total phenolic compounds.

* *p* < 0.05.

A total of 56 different phenolic compounds were identified, but some were present in more than one species. 14 phenolic compounds were identified in TO and CO, 20 in AM, 10 in AR and 17 in CN, belonging to this set of 56 compounds. The main families of phenolic compounds determined were phenolic acids and flavonoids. In the case of phenolic acids, they were essentially represented by caffeic acid derivatives, accounting for up to 26 identified compounds, especially several isomers of caffeoylquinic acid (CQA) and caffeoyl‐2,7‐anhydro‐3‐deoxy‐2‐octulopyranosonic acid (CDOA), which have been thoroughly reported in previous studies (Barroso et al. 2016; Padilla‐González et al. 2020). Compound **14** was identified as caffeic acid for the similarity of its MS fragmentation, UV spectra pattern, and retention time when compared against the available commercial standard. Peaks **1**, **5**, **7**, **15**, **35**, **38**, **43**, and **46** were identified as CDOA derivatives according to previous identification works. Specifically, a similar pattern of MS and UV spectra characteristics was observed in other species of the Asteraceae family, such as *Erigeron breviscapus* (Vant.) Hand.‐Mazz. and *C. nobile* (Caleja et al. 2015; Zhang et al. 2007). Peaks **2**, **3**, **4**, **8**, **19**, **27**, **36**, **40**, **42**, **49**, **50**, and **54** corresponded to mono‐, di‐, tri‐, and tetra‐CQAs. They were identified based on the relative retention times and the pattern of MS^2^ fragmentation, using the hierarchical key described by Clifford et al. (Clifford et al. 2003; Clifford, Knight, and Kuhnert 2005). Specifically, Compounds **2, 4**, and **8** (*m/z* 353, [M–H]^−^) were identified as 3‐CQA, 4‐CQA, and 5‐CQA, respectively. Their fragmentation behavior was consistent with literature data and with those previously reported by other authors (Clifford et al. 2003; Clifford, Knight, and Kuhnert 2005). Peaks **2** and **3** were identified as *cis‐* and *trans*‐3‐*O*‐CQA, respectively, based on the elution order described in previous studies (Barros et al. 2012; Clifford et al. 2008). Compounds **36, 40, 42, 49**, and **50** ([M‐H]^−^ at *m/z* 515) corresponded to *O*‐dicaffeoylquinic acid and their isomers, producing an MS^2^ peak at *m/z* 353 from the loss of caffeoyl moieties [M‐H‐162]^−^, whose subsequent fragmentation produced characteristic product ions at *m/z* 191, 179, 173, and 135. Compounds **40** and **42** were attributed to *cis‐* and *trans*‐3,5‐*O*‐diCQA, respectively, according to the elution order (Barroso et al. 2014). Similarly, Peaks **49** and **50** were tentatively identified as *cis*‐ and *trans*‐4,5‐*O*‐dicaffeoylquinic acid, based on the elution pattern previously described (Masike et al. 2018). Peaks **19** ([M–H]^−^ at *m/z* 839) and **27** ([M–H]^−^ at *m/z* 677) were assigned to 1,3,4,5‐tetra‐*O*‐CQA and 1,4,5‐*O*‐tricaffeoylquinic due to the presence of the MS^2^ peak at *m/z* 515 and 353, resulting from the losses of three and four caffeoyl moieties (−162 u, [M‐caffeoyl‐H]^−^), respectively. CQA derivatives have been largely reported by several authors in different species from the Asteraceae family (Dias et al. 2014; Petropoulos et al. 2019; Yılmaz et al. 2021). Peak **54** ([M‐H]^−^ at *m/z* 499), assigned as 4‐caffeoyl‐5‐*p*‐coumaroylquinic acid, lost its caffeoyl residue first and produced *m/z* 163 and 119 base peaks, respectively, as previously determined by Jaiswal, Deshpande, and Kuhnert (2011). Compound **17** ([M − H]^−^ at *m/z* 367) was identified as feruloylquinic acid, taking into account its pseudo‐molecular ion and MS^2^ fragmentation similar to the CQA previously described. Peaks **6** ([M‐H]^−^ at *m/z* 341) and **16** ([M‐H]^−^ at *m/z* 473) were assigned to caffeic acid hexoside and hexosyl‐pentoside, respectively. The product ion at *m/z* 179 ([caffeic acid‐H]^−^), resulting from the losses of 162 u and 294 u (hexosyl and hexosyl‐pentoside residue, respectively), provided the basis for their identification, along with their UV spectra characteristic, which belong to caffeic acid derivatives. The presence of caffeoyl‐hexoside‐methylglutarate ([M‐H]^−^ at *m/z* 469) was detected as Peak **45**. This compound revealed two fragmentation ions at *m/z* 307 ([M‐caffeoyl‐H]^−^) and 179 ([M‐hexosyl + methylglutarate‐H]^−^), which confirmed this identification, as already indicated elsewhere for CN extracts (Guimarães et al. 2013). The fragmentation pattern and elution order of Peaks **10** and **12** ([M–H]^−^ at *m/z* 473) allowed them to be assigned to *cis*‐ and *trans*‐chicoric acid (dicaffeoyltartaric acid), respectively (Petropoulos et al. 2018). These isomers were also reported by other authors in *T. officinale* and other Asteraceae plant extracts, with similar fragmentation (Petropoulos et al. 2019; Schütz et al. 2005), based on their product ion at *m/z* 311, resulting from the loss of a caffeoyl moiety (162 u).

In the case of flavonoids, 22 different compounds were found in these Asteraceae extracts. Among the flavonoids, two flavones were the main derivatives present, namely luteolin and apigenin derivatives, followed by flavonols, mainly isorhamnetin and quercetin glycosides derivatives. Peaks **9** (apigenin‐7‐*O*‐glucoside), **26 (**quercetin‐3‐*O*‐rutinoside), **34** (luteolin‐7‐*O*‐ deoxyhexosyl‐hexoside), and **39** (luteolin‐7‐*O‐*glucoside) were identified through the similarity of the MS fragmentation, UV spectra, and retention times when compared against certified standards.

Regarding flavones, Peaks **11** ([M–H]^−^ at *m/z* 573) and **13** ([M–H]^−^ at *m/z* 593), **18** and **52** ([M–H]^−^ at *m/z* 563), and **31** ([M–H]^−^ at *m/z* 577) were identified as apigenin derivatives, according to their UV and mass spectra characteristics and the fragmentation patterns previously described by other authors (Dias et al. 2013; Ferreres et al. 2003; Ҫiҫek et al. 2021). All these compounds generated the following MS^2^ fragments: *m/z* 473 and 383, corresponding to the respective loss of 120 u and 90 u, and a *m/z* 353 that was consistent with the apigenin aglycone with some sugar residues; therefore, these compounds were identified as different *C*‐hexosyl apigenins, as previously reported by Ferreres et al. (2003). Nevertheless, Peak **55** presented ([M‐H]^−^ at *m/z* 473) a unique MS^2^ fragment at *m/z* 269 ([M‐apigenin‐H]^−^), showing a loss of −204 u, 42 u higher than Peak **9**, which may be due to an acetyl group. Therefore, this compound was tentatively attributed to apigenin‐*O*‐acetyl‐hexoside.

Peaks **23** and **39** ([M–H]^−^ at *m/z* 447) are flavones *O*‐glycosides that generated MS^2^ fragment ions at *m/z* 285. Thus, they were assigned as luteolin‐*O*‐hexoside and luteolin‐*O*‐glucoside, both showing a loss of 162 u. However, Peak **20** presented the same pseudo‐molecular ion as Peaks **23** and **39** with an MS^2^ ion at *m/z* 357 [M − H − 90], indicating a mono‐*C*‐glucoside link. Moreover, the MS^2^ spectra showed no loss of a water molecule, which is characteristic for *C*‐6‐ glycosides, being assigned as luteolin‐*C*‐glucoside (Ferreres et al. 2003).

Peaks **47** ([M–H]^−^ at *m/z* 709), **51** ([M–H]^−^ at *m/z* 533), and **56** ([M–H]^−^ at *m/z* 579) released the same MS^2^ fragment at *m/z* 285 ([M‐86‐176‐162]^−^, [M‐162‐86]^−^, [M‐162‐132]^−^, associated with the loss of malonylglucuronyl‐hexoside, malonyl‐hexoside, and pentosyl‐hexoside residues, respectively), thus being tentatively identified as luteolin‐*O*‐malonylglucuronyl‐hexoside, luteolin‐*O*‐malonylhexoside, and luteolin‐*O*‐pentosylhexoside, respectively.

With respect to flavonols, Peaks **21** ([M‐H]^−^ at *m/z* 755), **30** ([M‐H]^−^ at *m/z* 609), and **41** ([M‐H]^−^ at *m/z* 505) released a unique MS^2^ fragment at *m/z* 301, which corresponds to the quercetin aglycone, with the loss of deoxyhexosyl‐rutinoside, deoxyhexosyl‐hexoside, and acetyl‐hexoside moieties ([M‐146‐162]^−^, [M‐146‐162]^−^, and [M‐42‐162]^−^, respectively). These were tentatively identified as quercetin‐3‐*O*‐deoxyhexosyl‐rutinoside, quercetin‐deoxyhexosyl‐hexoside, and quercetin‐3‐*O*‐acetyl‐hexoside. Peak **33** ([M–H]^−^ at *m/z* 477) showed similar fragments and was determined as quercetin‐3‐*O*‐glucuronide.

Peak **24** ([M–H]^−^ at *m/z* 479) released an MS^2^ fragment at *m/z* 317 ([M‐162]^−^), which corresponds to the myricetin aglycone, and was assigned as myricetin‐3‐*O*‐glucoside. Peak **25** ([M‐H]^−^ at *m/z* 609) presented an MS^2^ fragment ion at *m/z* 285, corresponding to the kaempferol aglycone ([M‐162‐162]^−^ loss of two hexosyl moieties). The loss of the two sugar moieties pointed out that they were linked as a disaccharide, being identified as a kaempferol‐*O*‐di‐*O*‐hexoside.

The remaining compounds corresponded to flavonoids, namely *O*‐methylated flavonols, Peaks **28**, **37**, and **48** ([M–H]^−^ at *m/z* 623), **29** and **32** ([M–H]^−^ at *m/z* 769), **44** ([M–H]^−^ at *m/z* 609), and **53** ([M–H]^−^ at *m/z* 519) were identified as isorhamnetin derivatives, considering the product ion at *m/z* 315 and the UV spectra (*λ*_max_ 352–358 nm). Peaks **28**, **37**, and **48** presented the loss of deoxyhexosyl‐hexosyl moiety ([M‐308]^−^). These compounds were tentatively identified as isorhamnetin‐*O*‐deoxyhexoside‐hexoside. Peaks **29** and **32** showed the same pseudo‐molecular ion ([M − H]^−^ at *m/z* 769) and presented the loss of deoxyhexoside‐rutinoside ([M‐176‐308]^−^), which may indicate their identification as different isorhamnetin‐*O*‐deoxyhexoside‐rutinoside isomers. Similarly, Peak **44** showed a −294 u, identified as the loss of a pentosyl‐hexoside moiety; thus, it may be tentatively identified as an isorhamnetin‐*O*‐pentosyl‐hexoside. Peak **53** was tentatively identified as isorhamnetin‐*O*‐acetylhexoside according to the pseudo‐molecular ion [M‐H]^−^ at *m/z* 519 and the MS^2^ fragment released at *m/z* 315 [M‐H‐42‐162]^−^, corresponding to the loss of an acetyl‐hexoside moiety. These compounds were already described by Miguel et al. in CO (Miguel et al. 2016). Finally, Peak **22** ([M‐H]^−^ at *m/z* 463) presented an MS^2^ fragmentation, corresponding to the loss of a glucuronide moiety [M‐176]^−^, producing an ion at *m/z* 287, which led to the tentative identification of an eriodictyol‐*O*‐glucuronide.

### 3.2 Quantification and Multivariate Statistics on the Phenolic Profile of Asteraceae Extracts

Following compound detection and identification, phenolic compounds were quantified individually, as well as the total content of phenolic acids (TPA), flavonoids (TFC), and phenolic compounds (TPC; Table 1). In general, the AR extract exhibited the highest content of phenolic acids, with 95.9 ± 1 mg/g extract, principally due to the high concentrations of 3,5‐*O*‐diCQA (45 ± 1 mg/g extract) and 5‐*O*‐CQA (20.3 ± 0.3 mg/g) (Table 1). Otherwise, AC extracts revealed a high content of phenolic acids, showing a 10% lower concentration than AR extracts. On the other hand, CN extracts showed a 75% lower concentration in comparison with AR, whereas TO and CO presented the lowest contents of phenolic acids, ranging from 13 to 17 mg/g extract, approximately. It is important to note that phenolic acid content has been reported to be highly influenced by the chemical nature of extraction solvents, as largely reported in AM and other Asteraceae species (Abou Baker 2020). Regarding flavonoids, CN extracts showed the highest flavonoid content (76 ± 1 mg/g), with luteolin‐*O*‐pentosyl‐hexoside and apigenin‐6,8‐*C*‐diglucoside being the most prevalent flavonoids, at concentrations of 50 ± 0.5 and 19 ± 1 mg/g, respectively. As it occurred with phenolic acids, AC extracts exhibited a lower amount, but with a relevant flavonoid content (50 ± 1 mg/g), whereas AR showed a 90% lower flavonoid content than CN (Table 1). Finally, concerning the total phenolic content, AC showed the highest values (136 ± 1 mg/g), whereas AR exhibited the second highest content (119 ± 1 mg/g), mostly due to its high phenolic acid concentrations. CN showed a moderate phenolic content (100 ± 1 mg/g) with the highest concentration of flavonoids (76 ± 1 mg/g). CO and TO showed the lowest values, ranging from approximately 19 to 29.2 mg/g. As stated earlier, the production of phenolic compounds by Asteraceae species is highly dependent on physiological, environmental, and processing factors, so the application of multivariate statistics is required to decipher the potential of phenolic compounds in the discrimination of the Asteraceae species involved in this study.

**FIGURE 2:**
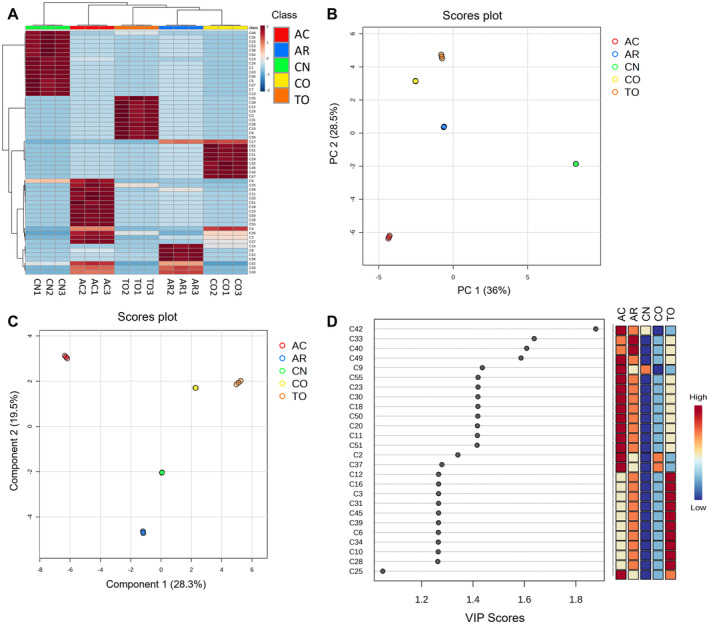
Multivariate statistics on the phenolic profile of Asteraceae extracts. For compound identification, refer to the compounds' peaks listed in Table 1. (A) Hierarchical cluster analysis (Euclidean distance; Ward's linkage algorithm). (B) Principal component analysis. (C) Partial least squares model. (D) Discriminant markers projected from the PLS model, represented by variable important in projection markers (VIPs; VIP score cut‐off = 1.0).

**FIGURE 3:**
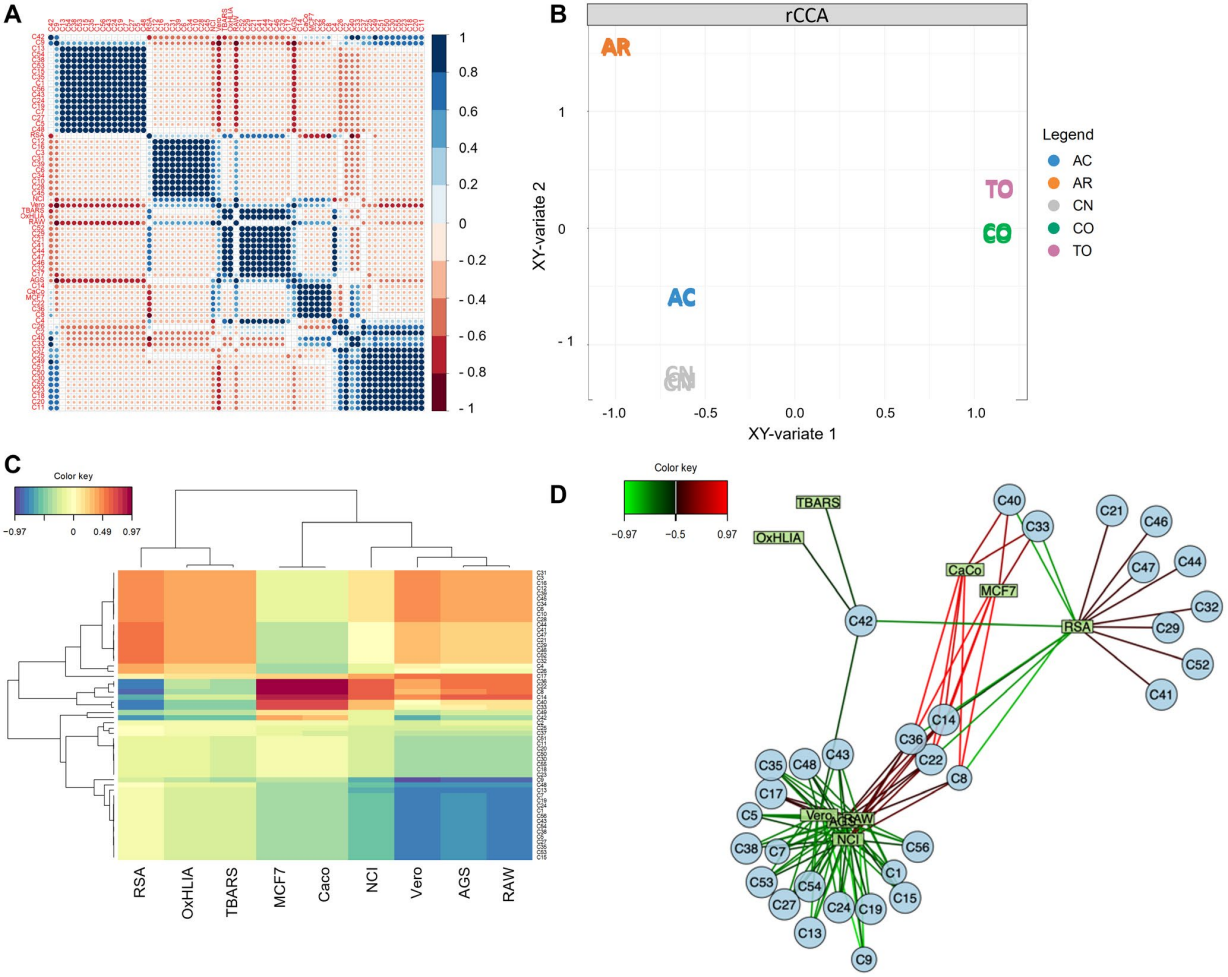
Integrative computational chemometrics of Asteraceae extracts between phenolic compounds and in vitro biological activities. (A) Pearson's correlation diagram. (B) Regularized canonical component analysis. (C) Integrative cluster map based on correlation coefficients (Euclidean distances). (D) Integrative network based on correlation coefficients (correlation coefficient cut‐off = 0.5).

The results for the multivariate statistics on Asteraceae phenolic extracts are shown in Figure 2, combining an unsupervised analysis through hierarchical cluster analysis (HCA; Figure 2A) and PCA (Figure 2B). Considering their phenolic profile, CN was found to independently cluster with respect to the rest of the species (Figure 2A), which was further confirmed by PCA, supported by a high percentage of variation (PC1 = 36%; PC2 = 28.5, Figure 2B). In contrast, CO, TO, and AR were found to exhibit a similar profile, making part of the same subcluster and being grouped according to PCA, while AC also presented a specific intermediate phenolic signature (Figures 2A and 3B). Furthermore, the results for the supervised PLS modeling show a similar outcome, discriminating all species according to the first component (28.3%), whereas the second component (19.5%) suggested a slight similarity among CO and TO (Figure 2C). These results partially agree with the phylogenetic structure of the Asteraceae family, since all the species belong to the Asteroideae subfamily, with the exception of TO, which belongs to the Cichorioideae subfamily (Panero and Crozier 2016), thus suggesting that the phenolic profile plays an important role in Asteraceae phylogenesis.

The discrimination reported by the PLS model was projected into the discriminant features identified as VIP markers (Figure 2D). Among them, AC was associated with the most discriminant features, justifying its specific profile, mostly led by apigenin glycosides (C9, C11, C18, C51, and C55), luteolin glycosides (C20, C23, and C51), and CQA derivatives (C2, C42, and C49). These results are in accordance with previous studies performed on AC, revealing that apigenin and luteolin were the most abundant flavonoids in this species (Ayoobi et al. 2019), while 5‐*O*‐CQA was reported as the major phenolic acid in ethanolic AC extracts (Craciunescu et al. 2012). Moreover, TO extracts also exhibited a wide range of associated discriminant features, represented by caffeic acid derivatives (C3, C6, C10, C12, C16, and C45), among which *cis*‐ and *trans‐*chicoric acid were featured. These compounds are attributed to the Cichorioideae subfamily (Panero and Crozier 2016), to which TO belongs. In the case of AR extracts, C33 and C40 were found as discriminant, identified as quercetin 3‐*O*‐glucuronide and *cis*‐3,5‐*O*‐diCQA, which have previously been highlighted as the most prevalent flavonoid and phenolic acid in this species (Ganzera et al. 2008).

### 3.3 Determination of the In Vitro Antioxidant Activity of Asteraceae Extracts

Table 2 depicts the results obtained for the determination of the antioxidant activity of Asteraceae extracts in terms of RSA, TBARS, and OxHLIA assays. The antioxidant bioactivity has a strong multifactorial behavior. Therefore, it is crucial to assess the antioxidant capacity through different assays to better understand which mechanisms are involved in each plant extract. In fact, oxidation occurs in multiple ways in living organisms, commonly starting from the production of reactive oxygen species (ROS), which generate harmful free radicals that may attack several subcellular structures, such as proteins and membrane phospholipids, giving rise to the onset of oxidative stress (García‐Pérez et al. 2019). Therefore, the parallel determination of different antioxidant mechanisms is essential to unravel the potential of plant extracts.

**TABLE 2:** Antioxidant, cytotoxic, and anti‐inflammatory activities of Asteraceae extracts.

	*Taraxacum officinalis*	*Calendula officinalis*	*Achillea millefolium*	*Arnica montana*	*Chamaemelum nobile*	Positive controls
Antioxidant activity (IC_50_, μg/mL)						Trolox
RSA	20 ± 2c	24 ± 1d	13 ± 1b	5.9 ± 0.6a	15 ± 1b	19 ± 1
TBARS	35 ± 4b	244 ± 16c	13 ± 1a	16 ± 1a	19 ± 1ab	5.4 ± 0.3
OxHLIA (Δ*t* 60 min)	154 ± 6d	n.a.	43 ± 1b	2.5 ± 0.2a	102 ± 2c	19.7 ± 0.4
OxHLIA (Δ*t* 120 min)	270 ± 11d	n.a.	89 ± 1b	15.4 ± 0.9a	193 ± 2c	41.8 ± 0.5
Cytotoxic activity (GI_50_, μg/mL)						Ellipticine
AGS	225 ± 9c	310 ± 17d	87 ± 3b	337 ± 17e	10.3 ± 0.3a	1.23 ± 0.03
Caco‐2	136 ± 13c	198 ± 9d	42 ± 4b	> 400	13 ± 1a	1.21 ± 0.02
MCF‐7	191 ± 14c	189 ± 12c	125 ± 2b	> 400	36 ± 3a	1.02 ± 0.02
NCI‐H460	> 400	254 ± 13c	124 ± 6b	> 400	54 ± 5a	1.01 ± 0.01
VERO	> 400	> 400	173 ± 13b	> 400	59 ± 2a	1.14 ± 0.06
Anti‐inflammatory activity (IC_50_, μg/mL)						Dexamethasone
RAW 264.7	106 ± 6d	72 ± 6c	30 ± 3b	111 ± 10d	15.2 ± 0.1a	6.3 ± 0.4

*Note:* Different letters indicate statistically significant differences according to Tukey's HSD test (*α* = 0.05).

Abbreviation: n.a., no activity.

The results for RSA show that the AR extract possessed the highest antioxidant activity, with 5.9 ± 0.6 μg/mL, followed by AC and CN extracts, both being higher than the positive control (Trolox, 19 ± 1 μg/mL). It is noteworthy that, due to the heterogeneous composition of plant extracts, some synergistic and antagonistic effects may potentially occur, simultaneously contributing to their total antioxidant activity. This suggests a potential synergistic effect between the phenolic compounds present in these extracts. Indeed, other authors have also demonstrated the higher RSA efficiency of AR hydroethanolic extracts compared to that of Trolox (Craciunescu et al. 2012). According to the phenolic composition, AR extracts promoted the highest antioxidant activity, driven by the accumulation of phenolic acids. This hypothesis is reinforced by the properties attributed to caffeic acid derivatives, especially chlorogenic acid, which is widely found in this extract, as potent antioxidant compounds reported in in vivo models (Sato et al. 2011).

The results from TBARS determination showed that all extracts promoted a lower antioxidant activity than the positive control, Trolox. Among the different plant extracts, AC and AR promoted the best results, showing the lowest IC_50_ values: 13 ± 1 and 16 ± 1 μg/mL, respectively (Table 2), showing a parallel response than that determined for RSA. Our results were shown to be higher than those previously reported for AC infusions (> 70 μg/mL) (Dias et al. 2013), and in line with the results obtained for different alcoholic extracts from *Achillea* species, such as *Achillea eriophora* and *Achillea biebersteinii*, whose IC_50_ values ranged from 14 to 24 μg/mL (Varasteh‐Kojourian et al. 2017). Furthermore, the lipid peroxidation inhibitory properties of AR extracts, determined by the TBARS assay, were associated with the alleviation of oxidative stress in vivo, as recently demonstrated in UVB radiation–induced skin‐burned mice models (da Silva Prade et al. 2020).

With respect to the results for the OxHLIA assay, the findings were similar to those observed for RSA, as AR extracts promoted the highest antioxidant activity (2.5 ± 0.2 μg/mL), which was 87% more potent than that of Trolox (19.7 ± 0.4 μg/mL) 60 min after the oxidation onset. The similar results found for oxidative hemolysis and RSA can be explained by the methodological bases of both assays, since OxHLIA requires the presence of free radicals that act as initiators of the oxidative burst in sheep erythrocytes (Takebayashi et al. 2012). In this regard, as observed for RSA, it is suggested that the high content of phenolic acids found in AR extract (Table 1) is one of the major causes contributing to this potent antioxidant activity. Moreover, the same trend was observed for the results obtained after 60 and 120 min of oxidation, indicating that phenolic compounds from Asteraceae extracts act coordinately over an extended period (Table 2). Compared to other species belonging to the Asteraceae family, previous works indicated that both AR and AC extracts exhibit more potent antioxidant activity, as determined by the OxHLIA assay, than seed extracts of *Cynara cardunculus* (79 μg/mL). This activity was proportional to the presence of CQAs in AR and AM extracts (Mandim et al. 2020).

### 3.4 Determination of the In Vitro Cytotoxic Activity of Asteraceae Extracts

The cytotoxic activity of Asteraceae extracts was determined by the sulforhodamine B assay, and the reported results are shown in Table 2. As can be observed, all extracts showed lower cytotoxic activity compared to ellipticine, used as the positive control, whose GI_50_ values were similar across all the different cancer cell lines tested (1.01–1.23 μg/mL). Nevertheless, this compound was shown to strongly inhibit the growth of the non‐tumor Vero cell line, exhibiting a deleterious effect on healthy cell lineages. Such toxic effects are a major concern associated with this drug, and great efforts are currently underway to mitigate the side effects of ellipticine treatment, such as nanoparticle design and chemical synthesis of less toxic derivatives (Indra et al. 2019; Nishiyama et al. 2017). Regarding the extracts, CN exhibited, by far, the highest cytotoxic activity against all cell lines tested, followed by AM (Table 2). In contrast, the AR extract showed negligible cytotoxic activity, which contrasts with the antioxidant activity‐related results. Regarding cell lines, a differential sensitivity was reported, with AGS and Caco‐2 being the most affected cell lines, showing GI_50_ values of 10.3 ± 0.3 and 13 ± 1 μg/mL, respectively, when treated with the CN extract (Table 2). On the other hand, the Vero cell line showed the highest GI_50_ values. Indeed, AR, TO, and CO extracts did not show significant cytotoxicity toward this healthy cell line.

The reasons behind the cytotoxic activity reported for CN extract could potentially be due to the high concentration of flavonoids (76 ± 1 mg/g extract), with luteolin and apigenin glycosides being the most abundant compounds (Table 1). In this regard, the antiproliferative effects of luteolin on the human gastric AGS cancer cell line have already been demonstrated, showing a time‐ and dose‐dependent behavior, with luteolin promoting cell cycle arrest at the G2/M phase and inducing the expression of proapoptotic transcription factors, such as caspase genes, Bax, and p53 (Wu et al. 2008). Additionally, the same authors indicated that luteolin caused an improvement in the chemosensitivity toward anticancer drugs, such as cisplatin (Wu et al. 2008). In parallel, the effectiveness of apigenin derivatives against the AGS cell line has been reported, with a low GI_50_ value of 1.21 μg/mL (Sharma et al. 2019). On the other hand, apigenin was shown to induce similar effects on the Caco‐2 cell line to those of luteolin on AGS, causing cell cycle arrest and promoting the expression of proapoptotic proteins (DeRango‐Adem and Blay 2021). Furthermore, the MCF‐7 cell line was also affected by the CN extract, though to a lesser extent (36 ± 3 μg/mL), but it showed 56.3% higher cytotoxic activity than previous studies using these extracts. These previous studies also showed a moderate cytotoxic activity against other cancer cell lines, such as HCT‐15 colon carcinoma, HeLa cervical carcinoma, and HepG2 hepatocellular carcinoma cell lines (Guimarães et al. 2013). Overall, these results open up a broad perspective on the use of CN extracts as a natural and safer strategy for the development of bio‐based antitumor treatments, either as a natural source of anticancer drugs or as adjuvants in chemotherapy, due to the previously observed synergistic effects with different drugs (Z. Zhang et al. 2021).

### 3.5 Determination of the In Vitro Anti‐Inflammatory Activity of Asteraceae Extracts

The results of the anti‐inflammatory activity on LPS‐induced RAW264.7 macrophage cell line are shown in Table 2. It is clearly observed that these results follow the same trend as those for cytotoxic activity: dexamethasone, used as the positive control, showed the highest activity. Regarding Asteraceae extracts, CN extract exhibited the lowest IC_50_ values (15.2 ± 0.1 μg/mL), followed by AM (30 ± 3 μg/mL). Again, AR exhibited the lowest anti‐inflammatory activity, along with TO extract (Table 2). Consequently, the highest correlation between both bioactivities can be explained based on the carcinogenetic process, in which inflammation plays a critical role during the early stages (García‐Pérez et al. 2019). Therefore, the high flavonoid concentration found in CN and AM extracts is suggested to contribute to the high anti‐inflammatory activity of these extracts. The effectiveness of apigenin on LPS–induced macrophages has been widely reported in the previous literature, showing that this flavonoid inhibits the expression and secretion of pro‐inflammatory cytokines (CKs) at low concentrations (8 μg/mL), as observed for tumor necrosis factor‐alpha (TNF‐*α*) (Palacz‐Wrobel et al. 2017). In parallel, apigenin derivative‐rich *Manilkara zapota* extracts inhibited the production of other CKs in the same macrophage model, such as prostaglandin E2 (Kamalakararao et al. 2018). Likewise, luteolin is considered a potent natural anti‐inflammatory compound, as it has been identified as an inhibitor of macrophage‐mediated phagocytosis and NO production (Rakariyatham et al. 2018; Tian et al. 2021). Besides flavonoids, other authors have demonstrated the effectiveness of octulosonic acid derivatives from *C. nobile* extracts, which are largely identified in our extracts (Table 1), as promising anti‐inflammatory candidates, through the inhibition of ROS–mediated oxidative stress (Zhao et al. 2014). In general, these results provide insight into the potential of CN extract as a promising source for producing natural anti‐inflammatory compounds. Nevertheless, further studies involving in vivo models are required to assess the actual anti‐inflammatory activity of these extracts.

### 3.6 Determination of the In Vitro Antimicrobial Activity of Asteraceae Extracts

The results for the antimicrobial activity of Asteraceae extracts are shown in Table 3. Asteraceae extracts show a multifaceted effectiveness against the heterogeneous panel of microorganisms selected for this study. In addition, the MIC, MBC, and MFC values indicate that, in general, all plant extracts tested were more efficient than the positive controls, E211 and E224. Regarding the extracts, CO exhibited the highest overall antibacterial activity, with MIC values ranging from 0.25 to 0.5 mg/mL, whereas CO and CN showed the highest antifungal activity, with the same MIC values (0.25–0.5 mg/mL) (Table 3). Among the different Gram‐negative strains, *E. coli* showed the highest sensitivity to the tested extracts, with MIC values of 0.25–0.5 mg/mL for plant extracts and 0.5–1 mg/mL for positive controls, whereas *B. cereus* was the most sensitive Gram‐positive strain, with an MIC of 0.25 mg/mL for all plant extracts, which was half the concentration of E211 and eight times lower than that of E224. Moreover, CO extracts exhibited the same effectiveness against all the Gram‐positive strains tested (Table 3). With respect to fungal strains, *T. viride* exhibited the highest sensitivity to plant extracts, whereas the remaining strains showed the same MIC and MFC values for each extract, with TO and AM extracts having the lowest antifungal activity (Table 3).

**TABLE 3:** Antimicrobial activity of selected plants from the Asteraceae family. Values represent extract concentrations in mg/mL.

	*Taraxacum officinalis*		*Calendula officinalis*		*Achillea millefolium*		*Arnica montana*		*Chamaemelum nobile*		Controls			
											E211		E224	
Gram‐negative bacteria	MIC	MBC	MIC	MBC	MIC	MBC	MIC	MBC	MIC	MBC	MIC	MBC	MIC	MBC
*Escherichia coli*	0.5	1	0.25	0.5	0.5	1	0.5	1	0.5	1	1	2	0.5	1
*Salmonella typhimurium*	1	2	0.5	1	1	2	0.5	1	0.5	1	1	2	1	1
*Enterobacter cloacae*	0.5	1	0.5	1	1	2	0.5	1	0.5	1	2	4	0.5	0.5
Gram‐positive bacteria	MIC	MBC	MIC	MBC	MIC	MBC	MIC	MBC	MIC	MBC	MIC	MBC	MIC	MBC
*Staphylococcus aureus*	1	2	0.25	0.5	0.5	1	0.5	1	0.5	1	4	4	1	1
*Bacillus cereus*	0.25	0.5	0.25	0.5	0.25	0.5	0.25	0.5	0.25	0.5	0.5	0.5	2	4
*Listeria monocytogenes*	0.5	1	0.25	0.5	0.5	1	0.5	1	0.5	1	1	2	1	0.5
Fungi	MIC	MFC	MIC	MFC	MIC	MFC	MIC	MFC	MIC	MFC	MIC	MFC	MIC	MFC
*Aspergillus fumigatus*	2	4	0.5	1	2	4	0.5	1	0.5	1	1	2	1	1
*Aspergillus niger*	2	4	0.5	1	2	4	1	2	0.5	1	1	2	1	1
*Aspergillus versicolor*	2	4	0.5	1	2	4	0.5	1	0.5	1	1	2	0.5	0.5
*Penicillium funiculosum*	2	4	0.5	1	2	4	0.5	1	0.5	1	1	2	0.5	0.5
*Trichoderma viride*	0.25	0.5	0.25	0.5	1	2	0.5	1	0.25	0.5	1	2	0.5	0.5
*Penicillium verrucosum* var. *cyclopium*	2	4	0.5	1	2	4	0.5	1	0.5	1	2	4	1	1

Abbreviations: MBC, minimal bactericidal concentration; MFC, minimal fungicidal concentration; MIC, minimal inhibitory concentration.

The remarkable antimicrobial activity of *C. officinalis* has been extensively reported against a plethora of both pathogenic bacteria and fungi (Efstratiou et al. 2012; Farjana, Zerin, and Kabir 2014). Nevertheless, the previous results have been mostly determined using the agar diffusion method, rather than the microdilution method used in this study. Thus, the results are difficult to compare. However, according to our results, although CO extracts presented a low concentration of phenolic compounds, > 40% were represented by chlorogenic acid, which has been widely identified as a potent antimicrobial agent. Thus, this phenolic acid exhibits MIC concentrations of 0.02–0.04 μg/mL for Gram‐positive bacteria, such as *S. aureus* , and 0.02–0.08 μg/mL for Gram‐negative bacteria, including *E. coli* and *S. Typhimurium* (Lou et al. 2011). In parallel, chlorogenic acid derivatives, which are widely reported in these extracts, exhibit similar antimicrobial activity to that of the parental compound (Naveed et al. 2018). Furthermore, chlorogenic acid has also been reported as a potent antifungal compound, potentially due to its ability to disrupt the development of cell membranes (Sung and Lee 2010).

On the other hand, CN extracts were revealed to be effective antimicrobial agents, as these extracts are rich in flavonoids, as previously stated. Once again, there is substantial evidence regarding the antimicrobial activity of this medicinal plant, as demonstrated for *S. aureus* and *E. coli* , among many other strains (Bail et al. 2009; Ghaedi et al. 2015). Owing to their phenolic composition, the high concentration of flavonoids on CN extracts may be responsible for such bioactivity. Specifically, luteolin was reported as a powerful antibacterial compound, thanks to its antibiofilm properties, recently found for *S. aureus* and *L. monocytogenes* (Qian et al. 2020). Consequently, both CO and CN extracts can be regarded as promising sources of natural antibiotics, thanks to their high proportions of chlorogenic acids and flavonoids, respectively.

### 3.7 Integration of Phenolic Profile and In Vitro Biological Activity of Asteraceae Extracts Through Regularized Canonical Component Analysis

The integration of the phenolic profile and in vitro bioactivities was performed to determine the eventual interactions between the identified compounds on the functional traits, contributing to the establishment of cause–effect relationships of Asteraceae phytoconstituents (Figure 3). First, a linear correlation analysis was carried out to initially determine the potential patterns of association between phenolics and in vitro biological activities. It is noteworthy that in vitro biological activities were expressed as IC_50_ and GI_50_, which means that compounds showing a negative correlation with bioactivities play a positive role. The correlation results show the existence of five highly correlated blocks, with only two blocks exhibiting a positive influence on the reported bioactivities (Figure 3A), thus suggesting a significant interaction of several compounds on the functionality of Asteraceae extracts.

An integrative computational approach based on rCCA was further performed to characterize the functional properties of Asteraceae extracts according to their phenolic profile. The obtained rCCA model shows three independent groups, indicating that TO and CO exhibit similar functionality, as well as CN and AC extracts, whereas AR extracts were found to reflect a specific bioactive outcome (Figure 3B). This model was further combined with an integrative cluster analysis based on the correlation coefficients between phenolic compounds and bioactivities to explore the bioactive properties attributed to the phenolic extracts of Asteraceae species (Figure 3C), showing that phenolics coordinately exerted antioxidant activity, as observed for the first branch of the cluster, as well as for the cytotoxic and anti‐inflammatory activities that were grouped in the second subcluster. This clustering was comprehensively incorporated into a network considering only the statistically significant correlations to unravel the possible synergistic events between phenolic compounds on the in vitro biological activities reported in Asteraceae extracts (Figure 3D).

The integrative network reflects three major functional clusters (Figure 3D), highlighting the importance of *trans*‐3,5‐*O*‐diCQA (C42), as it was significantly correlated with all antioxidant activities, RSA, TBARS, and OxHLIA, identifying this compound as a relevant candidate for the antioxidant properties attributed to Asteraceae species. There is wide evidence on the antioxidant properties of *trans* 3,5‐*O*‐diCQA, since its antioxidant activity was found to be twice that attributed to its analogs and derivatives, thanks to the structural configuration of its multiple hydroxyl groups that can also induce an enhancement of antioxidant activity when interacting with other molecules (Makori, Mu, and Sun 2021). Moreover, it was assessed as a potent plant‐based intracellular ROS scavenger (Sun et al. 2018), thus justifying its correlation with the inhibition of lipid peroxidation and oxidative hemolysis. Following antioxidant activity, a heterogeneous cluster was also correlated with RSA (Figure 3D), containing only flavonoid glycosides, from which most were represented by isorhamnetin glycosides (C29, C32, C44, C47, and C52). Interestingly, these flavonoids were not identified as discriminants for Asteraceae species, suggesting that they are common antioxidant agents of the Asteraceae family, having previously been assessed as efficient scavengers of a wide range of radicals (Wang et al. 2023). Finally, a third cluster was reported, reflecting a significant correlation with the cytotoxic activity against Vero, AGS, and NCI cell lines, as well as with the anti‐inflammatory activity against RAW‐mediated NO production (Figure 3D). In particular, this cluster essentially contained three groups of phenolic compounds, mainly represented by CDOAs (C1, C5, C7, C15, C35, C38, C43, and C53), which were revealed as the major cytotoxic and anti‐inflammatory candidates of Asteraceae extracts. Little is known about the biological properties attributed to CDOAs, although they have previously been reported in some medicinal plants. This confers new interest in the investigation of Asteraceae species, focusing on their associated anticancer and anti‐inflammatory properties. Besides CDOAs, the same cluster also presented other groups of cytotoxic and anti‐inflammatory compounds to a lesser extent; for instance, CQAs (C19, C27, C48, and C54) and flavonoid glycosides (C9, C13, C24, and C56). Among flavonoids, apigenin and luteolin glycosides were reported to exert intense bioactivity, as previously discussed for the cytotoxic and anti‐inflammatory results. Similarly, CQAs have been found to develop a heterogeneous mechanism of anti‐inflammatory action, repressing the production of pro‐inflammatory CKs by LPS–induced cultured macrophages, as well as being proven as effective cytotoxic agents against a plethora of cancer cell lines, such as Kato‐III stomach cancer, DLD‐1 colon cancer, and HL‐60 promyelocytic leukemia cancer cell lines (Panero and Crozier 2016). Overall, these results support the evidence of CQAs and CDOAs as meaningful representatives of the cancer‐preventing and anti‐inflammatory properties of Asteraceae extracts, whose efficiency has been shown to depend on the number of caffeoyl moieties within their structure.

## 4 Conclusions

In this work, the application of multivariate statistics and rCCA–based integrative computational chemometrics was applied to the UHPLC‐DAD‐ESI/MS^2^ phenolic profile of Asteraceae ethanolic extracts to unravel the authenticity markers and characterize the functional properties of five key species within this family, which are largely applied in traditional medicine worldwide. The discriminant phenolic markers identified in the phenolic Asteraceae extracts provided by multivariate statistics highlighted apigenin glycosides as the most important markers of *Achillea millefolium* , along with luteolin glycosides and CQA derivatives. Meanwhile, *Taraxacum officinale* extracts were found to exhibit chicoric acids as authenticity markers, whereas quercetin 3‐*O*‐glucuronide and *cis*‐3,5‐*O*‐diCQA were identified as markers of *Arnica montana* . Considering the profile‐wide perspective, the phenolic‐rich Asteraceae extracts led to species discrimination following the phylogenetic distribution of this family. Moreover, the integration of phenolics with the reported biological activities indicated that *trans*‐3,5‐*O*‐diCQA was mainly correlated with the antioxidant activity of Asteraceae extracts based on cell‐based systems, followed by isorhamnetin glycosides to a lesser extent. In parallel, caffeoyl‐dehydro‐octulopyranosonic acids were mainly associated with the anti‐inflammatory activity of the extracts, as well as with the cytotoxic activity against AGS gastric cancer and NCI non‐small cell lung cancer cell lines. Overall, these findings pave the way for the quality and authenticity assessment of Asteraceae extracts, leading to the discovery of their health‐promoting properties and contributing to their application in various industrial fields, especially in the food industry.

## Author Contributions

**Pascual Garcia‐Perez:** formal analysis (equal), software (equal), writing – original draft (equal). **Paula Garcia‐Oliveira:** conceptualization (equal), formal analysis (equal), investigation (equal), writing – original draft (equal). **Tiane C. Finimundy:** data curation (equal), formal analysis (equal), investigation (equal). **Jose Pinela:** formal analysis (equal), investigation (equal). **Ricardo C. Calhelha:** formal analysis (equal), investigation (equal). **Marija Nenadić:** formal analysis (equal), investigation (equal). **Marina Soković:** formal analysis (equal), investigation (equal). **Jesus Simal‐Gandara:** resources (equal), supervision (equal), writing – review and editing (equal). **Lillian Barros:** resources (equal), supervision (equal), writing – review and editing (equal). **Miguel A. Prieto:** resources (equal), supervision (equal), writing – review and editing (equal).

## Conflicts of Interest

The authors declare no conflicts of interest.

## Data Availability

Data are available on request from the authors.
